# Blame or discovery? Walter Benjamin's *Jetztzeit,* Purdue Pharma LP and ‘our values and our historical understanding of psychiatrists’

**DOI:** 10.1192/bjb.2023.39

**Published:** 2024-04

**Authors:** George Ikkos

**Affiliations:** Royal National Orthopaedic Hospital, London, UK

**Keywords:** History of psychiatry, Walter Benjamin, now-time (*Jetztzeit*), Purdue Pharma LP, Sackler

## Abstract

This commentary explores issues of professional identity, fairness and discovery in the history of psychiatry in the light of Walter Benjamin's (1892–1940) philosophy of history, especially his concept of *Jetztzeit* (now-time) and the profession's relationship with the founder and owners of Purdue Pharma LP.

‘For it is rightly said that truth is the daughter of time and not authority.’

Francis Bacon, *Novum Organon*, 1620, Book I

‘It may be that continuity is mere semblance. But then precisely the persistence of the semblance of persistence provides it with continuity.’

Walter Benjamin, *The Arcades Project*, N19,1

‘The now of recognisability is the moment of awakening.’

Walter Benjamin, *The Arcades Project*, N18,4

In her scholarly, nuanced and clinically relevant paper^1^ Claire Hilton, the Royal College of Psychiatrists’ Historian in Residence, highlights our professional continuity with our predecessors and the vital need to study and learn from their history unencumbered by arrogance or prejudice towards them. Among case examples, she discusses psychiatrist Henry Cotton's systematic removal of thousands of Americans’ internal organs for ‘treatment’ of mental illness and psychiatrists’ participation in the Nazi ‘euthanasia’ programme to systematically exterminate 80 000 Germans with severe neuropsychiatric disabilities through gas asphyxiation. Consistent with her institutional position, Hilton presses repeatedly her legitimate anxiety lest psychiatrists be unfairly criticised or blamed.

Although fairness is undoubtedly a foundational value in both history and psychiatry, arguably the key driver in historical enquiry should be discovery rather than praise or blame, necessary though these are too. In his ‘Copernican revolution in historical perception’ Walter Benjamin (1892–1940) proposed that ‘Formerly it was thought that a fixed point had been found in “what had been”, and one saw the present engaged in tentatively concentrating the forces of knowledge on this ground. Now this relation is to be overturned, and what has been is to become the dialectical reversal- the flash of awakened consciousness [ … ] The facts become something that just now first happened to us, first struck us’^2^ (K1,2). *Jetztzeit*: the ‘now-time’ of recognisability!

Although theories of sepsis and the spread of tonsillectomy may help contextualise Cotton's behaviour and it was lauded by some contemporary medical authorities, including British, we agree it still stands condemned as hubristic and inhumane. Regardless, the key message today is not the blame we may or may not apportion. It is the insight that, especially when general medical discoveries make them seem plausible, unwarranted clinical optimism combined with self-confidence and interventional fervour make psychiatrists vulnerable to tragically flawed actions, including toxic therapeutics. This extends beyond psychiatry, of course. The actions of Nazi psychiatrists were evil and those of opioid analgesia prescribers in recent decades not. Nevertheless, in the context of US deregulation and Sackler family-owned Purdue Pharma's criminality,^3^ harmful prescribing by physicians has caused much higher numbers of preventable deaths.^4^ To patients, doctors’ actions matter more than their motivations, although both are important.

‘History decays into images, not into stories’ noted Benjamin^2^ (N11,4). ‘It is not that what is past casts light on what is present, or what is present on what is past; rather image is that wherein what has been comes together in a flash with the now in the form of a constellation’^2^ (N3.1). We must not let fear, empathy or cognitive dissonance obscure the view.^5,6^ If oftentimes the constellation flashes most luminously in the dark, due diligence demands we go there. Raymond Sackler KBE (1920–2017) was a Life Fellow of the American Psychiatric Association and today the Institute of Mental Health at University College London is led by its Sackler Chair, inaugurated in 2018.^7^ Meanwhile the Tate Gallery in London ‘has become the latest institution to quietly drop the Sackler name from its walls in the race to cut ties with the disgraced family’.^8^

To conclude, in researching and reflecting on the history of psychiatry we will do best if we attend to Walter Benjamin's ‘Definitions of basic historical concepts: Catastrophe – to have missed the opportunity. Critical moment – the status quo threatens to be preserved’^2^ (N10,2).

## Data Availability

Data availability is not applicable to this article as no new data were created or analysed in this study.

## About the author

**George Ikkos** is a consultant liaison psychiatrist in the Department of Psychiatry at the Royal National Orthopaedic Hospital, London, immediate past Chair of the Royal College of Psychiatrists’ History of Psychiatry Special Interest Group (HoPSIG), and former President of the Royal Society of Medicine's Pain Medicine and Psychiatry Sections. He is joint editor of *Mind, State and Society: Social History of Psychiatry and Mental Health in Britain 1960–2010*, which was runner up in the Association of American Publishers’ PROSE 2022 Awards, in the section History of Science, Medicine and Technology (available open access from https://doi.org/10.1017/9781911623793).
